# Diabetes Mellitus and Multidrug-Resistant Gram-Negative Bacterial Infections in Critically Ill COVID-19 Patients: A Retrospective Observational Study

**DOI:** 10.3390/diagnostics15101190

**Published:** 2025-05-08

**Authors:** Vasiliki Dourliou, Nikolaos Kakaletsis, Dafni Stamou, Antigoni Champla, Kalliopi Tsakiri, Dimitrios Agapakis, Triantafyllos Didangelos

**Affiliations:** 1 Department of Adult Intensive Care Unit, Ippokrateio General Hospital, 54642 Thessaloniki, Greece; stdafni@hotmail.com (D.S.); antigoni_h@windowslive.com (A.C.); kalliopi.tsakiri@gmail.com (K.T.); 2 Internal Medicine Unit, Ippokrateio General Hospital, Aristotle University of Thessaloniki, 54642 Thessaloniki, Greece; kakaletsisnikos@yahoo.gr; 3 Department of Internal Medicine, Aghios Pavlos General Hospital, 55134 Thessaloniki, Greece; dimagap@yahoo.gr; 4 Diabetes Center, 1st Propaedeutic Department of Internal Medicine, Aristotle University of Thessaloniki, AHEPA Hospital, 54636 Thessaloniki, Greece; didang@auth.gr

**Keywords:** diabetes mellitus, COVID-19, SARS-CoV-2, multidrug-resistant Gram-negative pathogens, ICU

## Abstract

**Background:** Diabetes mellitus (DM) is an independent risk factor for severe SARS-CoV-2 infection and is linked to higher incidences of infections and adverse outcomes in patients with DM. This study examines the association between DM and multidrug-resistant Gram-negative bacteria (MDR-GNB) in critically ill, intubated COVID-19 patients in the intensive care unit (ICU) and evaluates mortality rates and clinical factors contributing to unfavorable outcomes. **Methods:** This retrospective observational study included intubated COVID-19 patients diagnosed with secondary infections due to MDR-GNB. Patients were treated for acute respiratory distress syndrome (ARDS) in a tertiary care university hospital ICU between October 2020 and February 2022. Collected data included demographics, comorbidities, medication, and laboratory parameters including blood tests and culture samples. **Results:** Among 416 COVID-19 patients, 112 (26.9%) had T2DM. Cultures from lower respiratory tract specimens revealed a significantly higher likelihood of isolating *Acinetobacter baumannii* in patients with DM (OR: 2.18, 95% CI: 1.40–3.40, *p* < 0.001), and DM is an independent predictor of isolation *Acinetobacter baumannii* in bronchial secretions of COVID-19 intubated patients (OR: 2.046, 95% CI: 1.256–3.333. *p* < 0.004). DM was not significantly associated with differences in length of stay (LOS) until discharge or death (HR: 0.76, 95% CI: 0.51–1.12, *p* = 0.16; HR: 0.91, 95% CI: 0.70–1.19, *p* = 0.50) or 28-day ICU mortality (OR: 1.12, 95% CI: 0.52–2.41, *p* = 0.77). Age was linked to an increased 28-day mortality risk in patients with DM (OR: 1.10, 95% CI: 1.02–1.18, *p* = 0.011). **Conclusions:** In critically ill intubated COVID-19 patients, DM emerged as a significant and independent predictor for the isolation of *Acinetobacter baumannii* from bronchial secretions, highlighting a key link between DM and specific multidrug-resistant pathogens, even though no broader association with MDR-GNB-related secondary infections was observed.

## 1. Introduction

The COVID-19 pandemic has highlighted the complex relationship between DM and infections, following observations that patients with DM are more likely to develop severe COVID-19 and have higher mortality rates compared to those without DM [1]. Since the beginning of the pandemic, DM has been reported as a major comorbidity, ranking after hypertension and cardiovascular disease [2]. The COVID-PREDICT prospective cohort showed that the presence of hypertension, dyslipidemia, and DM progressively leads to an increased risk of short-term mortality in hospitalized patients with COVID-19, independent of age and sex [3]. Notably, among these comorbidities, diabetes had the strongest association with mortality and remained a significant predictor even after adjusting for confounding factors (as surrogates for coronary artery disease), whereas the effect sizes for hypertension and dyslipidemia were smaller [3]. Similar results have been reported by the CDC [4] and CDC Weekly in China [5]. A meta-analysis of 30 studies involving a total of 6.452 patients showed that DM was associated with increased mortality, severe disease, acute respiratory distress syndrome (ARDS), the need for mechanical ventilation, and disease progression in patients with COVID-19, and this association may be linked to an inflammatory response in patients [6]. Consistently, an Italian cohort study of hospitalized COVID-19 patients found an increased prevalence of hypertension and diabetes among non-survivors; however, only diabetes remained an independent predictor of mortality after adjustment for other comorbidities [7]. These findings reinforce the notion that diabetes, more than other common comorbidities, independently contributes to poor outcomes in COVID-19.

Regarding the effect of DM on the pathophysiology of COVID-19 infection, it appears that SARS-CoV-2 infectivity may be enhanced by hyperglycemia, which may promote rapid viral replication. Hyperglycemia appears to directly increase SARS-CoV-2 replication in vitro, and glycolysis may sustain this viral replication [8]. In addition, the impaired immune response to SARS-CoV-2 due to chronic hyperglycemia and the low-grade chronic inflammation associated with T2DM contribute to a worse outcome [9]. Furthermore, DM can affect the immune system through several mechanisms, including poor chronic glycemic control and acute hyperglycemia [10,11]. Oxidative stress, along with the production of adhesion molecules involved in tissue inflammation, combined with the interaction of SARS-CoV-2 with ACE-2 receptors expressed in pancreatic, adipose, and intestinal cells, may lead to disruptions in glucose metabolism and contribute to the worsening of COVID-19 outcomes [12,13,14].

Importantly, during the COVID-19 pandemic, a substantial proportion of patients developed acute respiratory failure requiring admission to the intensive care unit (ICU) and mechanical ventilation. The demand for critical care resources, particularly invasive ventilatory support, was unprecedented, especially during the early pandemic waves [15]. Critically ill COVID-19 patients are at high risk of developing a wide range of complications that can significantly affect their clinical outcome and overall prognosis. Common complications seen in these patients include acute kidney injury, liver dysfunction, cardiovascular events, and thrombosis [16,17,18,19]. Additionally, major complications of these patients are secondary infections [20] and sepsis, septic shock, and multiorgan failure [19]. According to a special report by the CDC in 2022 [21], during the first year of the pandemic there were significant increases in infections, including hospital-acquired infections, due to multidrug-resistant pathogens such as carbapenem-resistant Acinetobacter, Gram-negative beta-lactamase-producing strains (ESBLs), vancomycin-resistant Enterococcus (VRE), and antifungal-resistant Candida. In fact, hospital-acquired infections and deaths due to resistant pathogens both increased by at least 15% during the first year of the pandemic. More specifically, regarding MDR-GNBs, the rate of carbapenem-resistant Acinetobacter increased overall in 2020 compared to 2019 by 35%, and similarly the rate of CRE (Enterobacterales resistant to carbapenems) infections increased by 35%. The proportion of ESBL-producing Enterobacterales also increased from 2019 to 2020, by 32% in inpatient infections and by 7% in community-acquired infections. Finally, in 2020, the proportion of MDR Pseudomonas aeruginosa cases increased by 32% compared to 2019. Epidemiological surveillance data from the European Antimicrobial Resistance Surveillance System (EARSS) and the Greek System for the Surveillance of Antimicrobial Resistance (WHONET) indicate that antimicrobial resistance and nosocomial infections pose a significant challenge in Greece, where multidrug-resistant Gram-negative bacteria are endemic [22,23].

The primary aim of this observational, retrospective study was to assess the impact of DM on the incidence of MDR-GNB in critically ill, intubated patients with SARS-CoV-2 (COVID-19) infection in the intensive care unit (ICU). Additionally, this study examined the association between DM and both 28-day ICU mortality and length of ICU stay in intubated COVID-19 patients with secondary infections caused by MDR-GNB.

## 2. Materials and Methods

### 2.1. Study Design

We conducted a retrospective observational study on polymerase chain reaction (PCR)-positive COVID-19 patients admitted to the ICU between October 2020 and February 2022 in a tertiary-level care university hospital in Thessaloniki, Greece. Patients ≥ 18 years or older with confirmed COVID-19 infection who were admitted to the ICU for acute respiratory distress syndrome (ARDS) and who developed secondary infections caused by MDR-GNB—defined as infections caused by MDR-GNB that were confirmed by positive cultures obtained ≥48 h after ICU admission, consistent with CDC criteria for healthcare-associated infections [24,25]—were included in this study. Patients with co-infection at ICU admission (less than 48 h), those with positive cultures for MDR-GNB within the first 48 h following ICU admission, and patients with T1DM and gestational diabetes were excluded from this study. We considered our patients as infected with the most prevalent variant of each pandemic wave; in the second wave of the COVID-19 pandemic in Greece (ISO WEEK 45/2020—03/2021) with variant B.1.1.7 (Alpha), in the third wave (ISO WEEK 11/2021—22/2021) with variant B.1.617.2 (Delta), and from the fourth wave through the beginning of the fifth (ISO WEEK 30/2021—03/2022) with variant B.1.1.529 (Omicron) [26]. All patients were mechanically ventilated.

### 2.2. Data Collection

Patient-level information was systematically collected from hospital records and included demographics (age, gender), detailed medical history (type 2 diabetes mellitus, hypertension, dyslipidemia, coronary artery disease, chronic kidney disease, anemia, hyperuricemia, and other relevant comorbidities), prior medications (antidiabetic drugs including insulin, metformin, sulfonylureas, DPP-4 inhibitors, SGLT-2 inhibitors, GLP-1 receptor agonists, antihypertensive medications such as angiotensin receptor blockers [ARBs], ACE inhibitors, calcium channel blockers, beta-blockers, diuretics, statins, antithrombotic agents such as aspirin and clopidogrel, anticoagulants including NOACs, acenocoumarol), and clinical severity scores (APACHE-II) on ICU admission. Results of the initial laboratory tests performed immediately after admission to the ICU were also collected. Data regarding antibiotic therapy were not included in the current study. The primary outcome of our study was defined as 28-day ICU mortality. Therefore, for the specific mortality analysis, only patients hospitalized in the ICU for fewer than 29 days were included. This exclusion criterion was established to provide a consistent and standard time frame for evaluating and comparing patient outcomes, aligning with widely accepted benchmarks used in critical care studies.

### 2.3. Laboratory Methods

Nasopharyngeal swabs were processed for the detection of the SARS-CoV-2 virus by reverse transcription PCR from hospital-affiliated certified laboratories. Secondary infections recorded retrospectively in this study, according to the European Centre for Disease Prevention and Control (ECDC) definitions from 2017, included bloodstream infections (BSIs) (primary and catheter-related), ventilator-associated pneumonia (VAP), and urinary tract infections (UTIs) caused by MDR-GNB [24,25]. Multidrug-resistant (MDR) bacteria were defined as those exhibiting resistance to at least one antibiotic from at least three different antibiotic groups in ≥3 antibiotic categories. Microbial identification and antimicrobial susceptibility testing were conducted at the microbiology laboratory utilizing the automated Vitek 2 system (Bio-Merieux, Marcy l’Etoile, France) [27].

### 2.4. Statistical Analysis

Statistical analysis of the data was performed through IBM SPSS Statistics for Windows (Version 29.0. Armonk, NY, USA: IBM Corp. Released 2022). Nominal data are presented as frequencies and percentages (*n*; %), and continuous variables are presented as means and ±standard deviation and medians and interquartile ranges after assessments for normality using the Kolmogorov–Smirnov test (≥50 samples). Our data were not parametric; thus, Pearson’s Chi-Square test or Fisher’s exact test were used for comparisons of nominal variables, while the Mann–Whitney U test and Kruskal–Wallis test were used to compare continuous variables. Univariate and multivariate logistic regression analysis (ENTER method) was conducted to determine the independent prognostic value of clinical, laboratory, and epidemiological risk factors that were found to be significantly associated with the outcomes. The associations were expressed as an odds ratio (OR) with corresponding 95% confidence intervals (CIs). A Bonferroni correction was applied where appropriate. Differences in survival and length of stay (LOS) between the two groups were assessed using the Kaplan–Meier method, with statistical significance evaluated via the log-rank test. To quantify the effect of covariates on overall survival, a Cox proportional hazards regression model was used. Hazard ratios (HRs) are presented along with their respective 95% confidence intervals (CIs). All statistical tests were 2-sided, and a *p* value less than 0.05 was taken as the level of statistical significance.

## 3. Results

A total of 416 adult patients with confirmed COVID-19 infection who were admitted to the ICU between October 2020 and February 2022 for acute respiratory distress syndrome (ARDS) and who developed secondary infections caused by MDR-GNB were enrolled.

Overall, in our study, 112 out of 416 patients (26.9%) had DM. Regarding the general characteristics of the patients with DM, several observations can be made (Supplemental Table S1). These patients were, on average, older (68.8 ± 9.6 years) than those without DM (61.4 ± 12.8 years), with the 7.5-year difference being statistically significant (*p* < 0.001). The proportion of male patients with DM was lower than that of non-DM patients (52.7% vs. 66.4%, *p* = 0.010). Admission trends showed that during the second and fourth pandemic waves, DM patients were admitted to the ICU less frequently compared to non-DM patients (21.4% vs. 24% and 38.4% vs. 52.3%, respectively, *p* = 0.003). However, during the third wave, DM patients were admitted nearly twice as often as non-DM patients (40.2% vs. 23.7%, *p* = 0.003).

DM patients also had a significantly higher prevalence of comorbidities, including dyslipidemia (78.6% vs. 32.9%, *p* < 0.001), hypertension (71.4% vs. 41.4%, *p* < 0.001), coronary artery disease (22.3% vs. 8.9%, *p* < 0.001), hyperuricemia (12.5% vs. 3.9%, *p* < 0.001), and anemia (11.6% vs. 5.3%, *p* < 0.001). They were more likely to be on statins (70.7% vs. 28%, *p* < 0.001), antithrombotic therapies such as aspirin (35.7% vs. 10.9%, *p* < 0.001), and clopidogrel (17.9% vs. 6.9%, *p* < 0.001), as well as NOACs (8.9% vs. 3.3%, *p* = 0.017). In terms of antihypertensive therapy, DM patients were more frequently prescribed angiotensin receptor blockers (49.1% vs. 22.7%, *p* < 0.001), diuretics (45.5% vs. 18.4%, *p* < 0.001), beta-blockers (38.4% vs. 25.3%, *p* = 0.009), calcium channel blockers (37.5% vs. 20.4%, *p* < 0.001), and aldosterone antagonists (4.5% vs. 0.7%, *p* = 0.007).

Clinically, at admission, DM patients presented with more severe conditions, evidenced by higher APACHE-II scores (14 ± 4 vs. 12 ± 3, *p* < 0.001) and more frequent acute kidney injury (25.9% vs. 14.9%, *p* = 0.010). They also had higher creatinine levels (1.3 ± 1.1 mg/dL vs. 1.0 ± 0.7 mg/dL, *p* = 0.003) and a lower estimated glomerular filtration rate (66.3 ± 28.8 mL/min vs. 81.6 ± 28.5 mL/min, *p* < 0.001). Furthermore, blood glucose levels were significantly elevated in DM patients, both at admission (231.5 ± 80.5 mg/dL vs. 163.2 ± 54.7 mg/dL, *p* < 0.001) and in their mean fasting glucose levels during their ICU stay (197.3 ± 46.1 mg/dL vs. 143.5 ± 38.3 mg/dL, *p* < 0.001). While DM patients experienced slightly longer hospital stays (20 ± 16 days vs. 18.1 ± 12.9 days, *p* = 0.493) and higher 28-day mortality (55.4% vs. 50.7%, *p* = 0.395), these differences were not statistically significant. Readmission rates were similar between the groups (5.4% vs. 6.6%, *p* = 0.648).

Regarding the MDR-GNB responsible for secondary infections in intubated COVID-19 patients with or without diabetes mellitus, as analyzed in Supplemental Table S2, no statistically significant differences were observed. From bronchial secretion cultures, only *Acinetobacter baumannii* was identified at a significantly higher rate in DM patients (48.2% vs. 29.9%, *p* < 0.001). The second most frequent isolated pathogen *Klebsiella pneumoniae*, was isolated at a similar rate in both subgroups (~17.3%), and the third most frequent, *Pseudomonas aeruginosa*, showed no statistically significant differences.

As for the most frequently MDR-GNB isolated from blood cultures, *Acinetobacter baumannii* and *Klebsiella pneumonia* were the most commonly identified pathogens, each isolated in approximately 20% of intubated patients, with no significant differences between patients with and without DM. Similarly, as in bronchial secretion cultures, *Pseudomonas aeruginosa* was the third most frequently isolated pathogen from blood cultures, but this difference was not statistically significant (6.3% vs. 3.6%, *p* = 0.289).

Regarding the most common MDR-GNB identified from central venous catheter tip and urine cultures, *Acinetobacter baumannii* and *Klebsiella pneumoniae* were once again the most frequent pathogens identified, with their proportions in DM patients not differing from those without DM.

Regarding patients with DM and their clinical outcomes in the present study (Table 1), among the 112 patients with DM, who represented 26.9% of the total study population, 86 were hospitalized in the ICU for fewer than 29 days, of whom 62 died (72.1%). The DM patients who died were significantly older than those who survived (71.6 ± 6.6 vs. 62.8 ± 14.5 years, *p* = 0.002). No significant differences were observed between the two groups in terms of comorbidities or pre-intubation antithrombotic and antihypertensive treatment. However, a higher proportion of deceased patients had been on oral antidiabetic therapy (71% vs. 41.7%, *p* = 0.021), while a lower proportion had received no antidiabetic treatment (3.2% vs. 20.8%).

Deceased DM patients presented with more severe clinical profiles, including higher APACHE-II scores [14.5 (7) vs. 13 (4), *p* = 0.010], more frequent acute kidney injury (35.5% vs. 12.5%, *p* = 0.035), and elevated creatinine levels (1.5 ± 1.4 vs. 1.1 ± 0.6 mg/dL, *p* = 0.045). Although blood glucose levels at admission and mean fasting glucose during the ICU stay did not differ significantly between survivors and non-survivors, inflammatory markers were markedly higher among those who died. Specifically, they had elevated white blood cell counts (15.3 ± 6.5 vs. 12.1 ± 5.3 K/μL, *p* = 0.033), serum ferritin levels (1280 ± 1684 vs. 956 ± 2339 ng/mL, *p* = 0.005), and procalcitonin levels (1.8 ± 4.7 vs. 0.2 ± 0.3 ng/mL, *p* = 0.030).

A univariate logistic regression analysis was performed to evaluate epidemiological factors, risk factors, and clinical/laboratory findings associated with 28-day mortality among all COVID-19 patients and patients with DM with LOS ≤ 28 days (Supplemental Table S3). As emerged from univariate logistic regression analysis across the entire study population, factors associated with 28-day mortality included age (OR: 1.06, 95% CI: 1.04–1.08, *p* < 0.001), hypertension (OR: 1.88, 95% CI: 1.19–2.96, *p* = 0.007), coronary artery disease (OR: 2.36, 95% CI: 1.09–5.10, *p* = 0.029), chronic obstructive pulmonary disease (COPD) (OR: 2.85, 95% CI: 1.06–7.68, *p* = 0.038), clinical severity upon admission (APACHE II score) (OR: 1.24, 95% CI: 1.15–1.35, *p* < 0.001), occurrence of acute kidney injury (OR: 2.92, 95% CI: 1.49–5.72, *p* = 0.002), elevated mean blood glucose levels (OR: 1.01, 95% CI: 1.00–1.01, *p* = 0.007), leukocytosis (OR: 1.07, 95% CI: 1.03–1.12, *p* < 0.001), increased serum creatinine (OR: 1.76, 95% CI: 1.14–2.72, *p* = 0.010), reduced eGFR (OR: 0.98, 95% CI: 0.98–0.99, *p* < 0.001), and increased ferritin (OR:1.00, 95% CI:1.00–1.00, *p* = 0.004). As far as patients with DM, age was independently associated with increased odds of death (OR: 1.10, 95% CI: 1.04–1.1, *p* = 0.002). Higher disease severity on ICU admission, as measured by the APACHE II score, was also significantly associated with mortality (OR: 1.21, 95% CI: 1.04–1.41, *p* = 0.013). The presence of acute kidney injury was associated with a nearly four-fold increase in the odds of death (OR: 3.85, 95% CI: 1.03–14.37, *p* = 0.045). Additionally, leukocytosis (OR: 1.10, 95% CI: 1.00–1.19, *p* = 0.041) and a decreased estimated glomerular filtration rate (eGFR) (OR: 0.98, 95% CI: 0.97–1.00, *p* = 0.047) were significantly associated with increased 28-day mortality risk.

A total of 10 variables (significance level of *p* < 0.05) were evaluated for their association with 28-day mortality in all ICU patients with LOS ≤ 28 days in the multivariate logistic regression model. To account for multiple comparisons, Bonferroni correction was applied, adjusting the statistical significance to *p* < 0.005. No variable remained as an independent predictor. As for 28-day mortality among patients with DM, four variables associated with 28-day mortality in the univariate logistic regression analysis at a significance level of *p* < 0.05 were considered eligible for inclusion in the multivariate logistic regression model. After applying Bonferroni correction for multiple comparisons (adjusted significance *p* < 0.0125), only age remained an independent predictor of 28-day mortality among patients with DM (OR: 1.10, 95% CI: 1.02–1.18, *p* = 0.011) (Table 2).

Univariate logistic regression analysis was also performed to assess the association between DM and MDR-GNB isolated from bronchial secretion cultures, blood cultures, central venous catheter tip cultures, and urine cultures (Table 3). In patients with DM, the likelihood of isolating *Acinetobacter baumannii* from bronchial secretion cultures was 2.2 times higher (OR: 2.18, 95% CI: 1.40–3.40, *p* < 0.001).

Specifically, a univariate logistic regression analysis was performed for *Acinetobacter baumannii* isolated from bronchial secretions, considering epidemiological factors, risk factors, and clinical/laboratory findings that may influence its identification in secondary respiratory infections (Supplemental Table S4).

Two variables associated with Acinetobacter baumannii isolation in bronchial secretions that were found in the univariate logistic regression analysis at a significance level of *p* < 0.05 were considered eligible for inclusion in the multivariate logistic regression model (Table 4). After applying Bonferroni correction for multiple comparisons (adjusted significance *p* < 0.025), only DM remained an independent predictor of isolation *Acinetobacter baumannii* in bronchial secretions of COVID-19 intubated patients (OR: 2.046, 95% CI: 1.256–3.333. *p* = 0.004).

To investigate the prognostic significance of MDR-GNB in overall mortality among patients with DM, a univariate analysis was performed (Table S5). As shown, in our study, MDR-GNB were not found to be associated with an increased risk of overall mortality in patients with DM.

**Figure 1:**
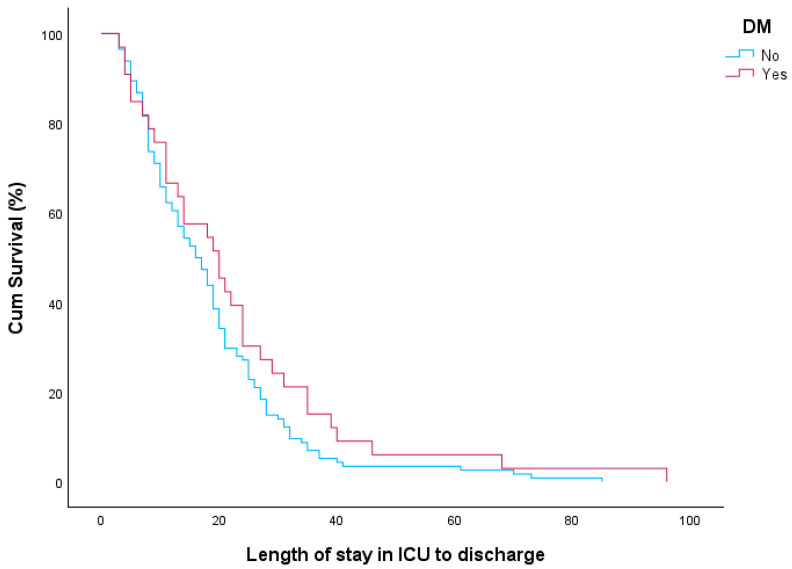
ICU length of stay until discharge for patients with and without DM.

As far as the effect of DM on ICU length of stay from admission to discharge, in our study, a survival analysis was performed using the log-rank test. The median time to ICU discharge was 20 days for patients with DM and 16 days for those without DM. However, this difference was not statistically significant (Log-rank test *p* = 0.149). The Kaplan–Meier curves presented in Figure 1 illustrate the ICU length of stay until discharge for patients with and without DM. Similarly, in the Cox proportional hazards model, DM was not found to be associated with an increased risk of prolonged ICU stay from admission to discharge. The hazard ratio for ICU discharge in patients with DM compared to those without was 0.755 (95% CI: 0.509–1.121, *p* = 0.163).

**Figure 2:**
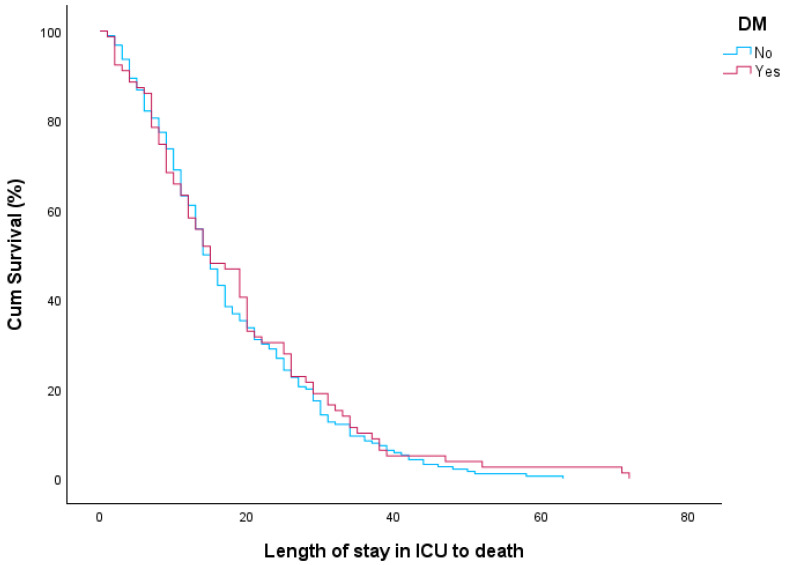
ICU length of stay until death for patients with and without DM.

A survival analysis using the log-rank test was subsequently performed to assess the impact of DM on ICU length of stay from admission to the adverse outcome of death (overall mortality). The median ICU stay until death was 15 days for patients with DM and 14 days for those without DM. However, this difference was not statistically significant (log-rank test, *p* = 0.488). The Kaplan–Meier curves presented in Figure 2 illustrate the duration of ICU stay until death for patients with and without diabetes mellitus. Finally, in the Cox proportional hazards model, diabetes mellitus was not found to significantly affect the hazard of prolonged ICU stay until death (HR: 0.913, 95% CI: 0.700–1.191, *p* = 0.502).

## 4. Discussion

In the present study, a total of 416 adult patients with confirmed COVID-19 infection who were admitted to the ICU between October 2020 and February 2022 for acute respiratory distress syndrome (ARDS) and who developed secondary infections caused by MDR-GNB were enrolled. Overall, in our study, 112 out of 416 patients (26.9%) had diabetes mellitus (DM). Comparatively, in a previous retrospective multicenter observational study of 90 patients with laboratory-confirmed SARS-CoV-2 pneumonia hospitalized in ICUs across eight reference hospitals in Greece from March 10 to April 13, 2020, during the first wave of the pandemic, hypertension was the predominant comorbidity (50%), followed by cardiovascular diseases (21.1%) and type 2 diabetes mellitus (18.9%) [28].

One of the initial observations for the present study concerns mortality among patients with DM. Since the beginning of the pandemic, several studies have indicated diabetes as a risk factor for mortality [29,30,31] and have shown that patients with DM and COVID-19 have worse outcomes than non-diabetic patients. In an early meta-analysis of 33 studies, Kumar et al. found more than twice the mortality risk and greater disease severity in COVID-19 patients with DM compared to those without DM [32]. Since diabetes frequently coexists with multiple other comorbidities, it remained unclear whether this association was independent of other factors. Nevertheless, this association was not identified in the present study. The 28-day mortality rate in the DM group was higher (55.4% vs. 50.7%), but the difference was not statistically significant to support diabetes as an independent risk factor for ICU mortality (OR: 1.12, 95% CI: 0.52–2.41, *p* = 0.769). The absence of a significant association between diabetes mellitus and 28-day ICU mortality in our study (OR: 1.12, 95% CI: 0.52–2.41, *p* = 0.769) contrasts with findings from several previous reports. A possible explanation for this discrepancy includes the limited statistical precision of our result, as reflected by the wide confidence interval, which may be attributed to the moderate sample size of diabetic patients or to inherent variability in clinical characteristics and comorbidities among critically ill COVID-19 populations. Future studies with larger cohorts could clarify this association more precisely. In a previous French study by Al-Salameh et al. [33]., involving 433 COVID-19 patients, 115 (26.6%) of whom had DM, diabetes was not associated with mortality but with ICU admission. Similarly, Orioli et al. [34] in Belgium found no significant differences in mortality rates when comparing short-term outcomes between COVID-19 patients with and without diabetes. Balta Başı et al. [35], in a retrospective observational study, found that diabetes did not influence ICU stay duration or mortality in patients admitted to the ICU due to COVID-19 pneumonia.

The mean age of DM patients in the current study was 68.8 years (range 48–89), statistically significantly higher than non-diabetic patients, confirming the difference observed in other studies [29,30]. Additionally, previous studies have already demonstrated older age as an independent risk factor contributing to worse COVID-19 prognosis [29,30]. In our analysis, older age emerged as an independent predictor of increased 28-day ICU mortality among diabetic patients (OR: 1.10, 95% CI: 1.02–1.18, *p* = 0.011). However, considering the frequent coexistence and strong correlation between age and diabetes, it is important to interpret this finding cautiously. Although our results indicate a significant statistical association, the observational nature of our study precludes direct inference of causality, and further prospective studies are needed to clarify the precise interplay between age, diabetes, and mortality in critically ill COVID-19 patients.

In addition to age, DM patients presented with more severe conditions, evidenced by higher APACHE-II scores (14 ± 4 vs. 12 ± 3, *p* < 0.001). It is noteworthy that although DM patients exhibited a higher mortality rate, this difference was not statistically significant. Importantly, DM patients also had significantly higher APACHE II scores at ICU admission, indicating a less favorable baseline prognosis compared to non-DM patients. Therefore, it is plausible that the increased mortality observed in DM patients may be attributed not solely to diabetes itself but rather to the associated complications and comorbid conditions frequently seen in this population, such as renal failure, ischemic heart disease, hypertension, and immune dysfunction [36,37].

Regarding other characteristics of the DM subgroup in this study, similar results emerged from a review of international studies (meta-analyses and observational studies)—a higher proportion of males (52.7%), with common comorbidities being dyslipidemia (78.6%), hypertension (71.4%), and coronary artery disease (22.3%). Many studies report hypertension as the most common comorbidity in COVID-19 patients, followed by diabetes and cardiovascular disease, confirming our findings, while Wang et al. [38] in a meta-analysis notably indicated that hypertension and diabetes are risk factors for worse COVID-19 prognosis.

Acute kidney injury (AKI) has been demonstrated as a severe complication of COVID-19, although its cause remains unclear. Direct cellular injury, cytokine storms, drug toxicity, and dehydration may be potential causes of renal damage in these patients [39]. In our study, AKI occurrence was significantly higher among DM patients (25.9% vs. 14.9%) compared to non-DM patients. However, in multivariate analysis, AKI was not a significant independent prognostic factor for poorer outcomes (death within 28 days). Elevated creatinine levels were also recorded in DM patients, similar to the study by Zhang et al. [31], while Shang et al. [30] reported different findings. According to Cecconi et al. [40], increased serum creatinine doubled the risk of deterioration (ICU admission or death) among hospitalized COVID-19 patients. Zhou et al. [36] reported up to 50 times higher AKI frequency among non-survivors.

Regarding laboratory characteristics, both admission glucose levels (213.5 vs. 163.2 mg/dL) and mean fasting glucose levels (197.3 vs. 143.5 mg/dL) were statistically significantly higher in DM patients compared to non-DM patients. However, in univariate analysis concerning 28-day mortality in DM patients hospitalized for less than 28 days, neither admission glucose nor mean morning glucose levels correlated with worse outcomes. Among COVID-19 patients, the prevalence of hyperglycemia does not significantly differ from the general population, suggesting that high glucose levels do not increase COVID-19 infection risk but significantly worsen outcomes [41,42,43,44]. Multiple clinical studies show that hyperglycemia is a significant risk factor for increased COVID-19 mortality and severity [31,45]. COVID-19 patients with uncontrolled hyperglycemia have a higher likelihood of ICU admission, with an estimated mortality rate approximately three times greater compared to patients without hyperglycemia [46]. Improved glycemic control may potentially improve clinical outcomes [47,48,49]. Several factors might account for the observed discrepancies between our findings and those reported by previous studies. These include variations in study design (e.g., retrospective vs. prospective studies), variations in sample sizes, differences in definitions of secondary infections and diagnostic criteria for MDR pathogens, and variability in the management of critically ill COVID-19 patients across different institutions and regions. Additionally, differences in local epidemiological patterns, antimicrobial stewardship practices, and infection control measures could have contributed to these inconsistencies. Further multicenter studies with standardized methodologies and larger cohorts are needed to confirm our findings and better understand these differences.

Regarding other laboratory parameters, white blood cell count, ferritin, procalcitonin, troponin, and D-Dimer were elevated in both DM and non-DM patients without significant differences. In a subgroup analysis based on outcomes among DM patients, those who died had higher white blood cell counts and higher ferritin and procalcitonin levels compared to survivors. However, in multivariate analysis, no statistically significant differences emerged. Cecconi et al. [40] reported that leukocytosis [white blood cell count > 10×10^9^/L], lymphocytopenia (lymphocyte count < 1 × 10^9^/L), and elevated levels of certain laboratory parameters such as procalcitonin, interleukin-6, serum ferritin, C-reactive protein, aspartate aminotransferase, serum creatinine, lactate dehydrogenase, fibrinogen, troponin-I, and D-Dimer were statistically significant predictors of clinical deterioration leading to ICU admission or death in hospitalized COVID-19 patients.

At the beginning of the pandemic, it was hypothesized that the increased risk of SARS-CoV-2 infection among diabetic or hypertensive patients might be due to increased ACE2 expression in patients receiving ACE inhibitors (ACEI) or angiotensin receptor blockers (ARBs) [50,51,52]. In this study, where hypertension represented the highest comorbidity, patients had been receiving ACEI/ARBs prior to SARS-CoV-2 infection. Specifically, diabetic patients had a higher percentage of antihypertensive treatment with ARBs (49.1% vs. 22.7%), while the percentage for ACE inhibitors was lower and did not significantly differ between the two subgroups (11.6% and 8.2%, respectively).

Another important aspect to consider in the context of COVID-19 mortality is the potential protective role of statins. Several studies have reported that patients who were on statin therapy prior to the onset of COVID-19-related respiratory failure had a lower risk of mortality, possibly due to the anti-inflammatory and immunomodulatory properties of statins [53]. Additionally, thromboprophylaxis with low-molecular-weight heparin has become a critical component of the management of hospitalized COVID-19 patients, given the well-recognized risk of thromboembolic complications. Although data on anticoagulant use during ICU hospitalization were not collected in our study, recent observational data from European ICUs have supported the safety and potential benefits of enhanced thromboprophylaxis strategies in critically ill COVID-19 patients [54].

Regarding multidrug-resistant (MDR) Gram-negative bacteria responsible for secondary infections in intubated COVID-19 patients, no statistically significant differences were observed between patients with or without diabetes, except for *Acinetobacter baumannii*, identified more frequently (48.2% vs. 29.9%) in bronchial secretions. Diabetes was found to be the only significant independent predictor for *Acinetobacter baumannii* isolation, with DM patients having twice the risk of developing secondary *Acinetobacter baumannii* infections compared to non-DM patients (OR: 2.046, 95% CI: 1.256–3.333, *p* = 0.004).

The literature review indicates that *Acinetobacter baumannii* is a significant risk factor for patients with type 2 diabetes (T2DM), associated with higher mortality rates among severely ill diabetic patients or those with elevated blood glucose levels and bacteremia in the ICU [55,56], and a higher risk of secondary infections in burns and diabetic foot infections [57,58,59]. In an experimental mouse pneumonia model, *Acinetobacter baumannii* caused increased pro-inflammatory cytokines, decreased neutrophil infiltration in lungs, and enhanced extrapulmonary spread [60]. This fact, combined with neutrophil dysfunction in T2DM patients (reduced early chemotaxis and compensatory hyper-inflammatory response) [61], may explain the potentially greater spread of *Acinetobacter baumannii* among T2DM patients. Perera et al. [62] found *Acinetobacter baumannii* in the serum of 23% of T2DM patients, independent of entry points, showing significant changes in cytokine profiles and broader inflammatory changes among those positive for *Acinetobacter baumannii* in serum and T2DM/periodontitis.

In the study by Polemis et al. [63], investigating the potential impact of COVID-19-related hospital practice changes on antimicrobial resistance (AMR) recorded by the AMR WHONET-Greece surveillance network, a notable increase in isolates from blood and respiratory cultures from ICU patients during the last six months of the study period (October 2020–March 2021) was found, primarily attributed to *Acinetobacter baumannii* isolation, increasing 1.24-fold in blood samples and 1.6-fold in bronchial secretions. In the current study, the most frequently identified MDR Gram-negative pathogen from blood cultures, bronchial secretions, and central venous catheter tips was *Acinetobacter baumannii*. *Klebsiella pneumoniae* was the second most frequent pathogen from blood cultures, bronchial secretions, and central venous catheter tips and the most frequent from urine cultures and rectal colonization. The rates of *Pseudomonas aeruginosa*, apart from bronchial secretions, were considerably lower compared to the other two MDR pathogens. Another retrospective cohort by Costa et al. [64], studying 191 COVID-19 ICU patients between March and May 2020, found the most frequent pathogens to be *Acinetobacter baumannii* (28.9%), *Pseudomonas aeruginosa* (22.7%), and *Klebsiella pneumoniae* (14.4%). Generally, from the literature review, several studies reported a decrease or no change in MDR pathogen colonization or infection rates in ICUs during the COVID-19 pandemic [65,66,67,68], while others noted significant increases in MDR infections [69,70,71] or an increase only in a specific subgroup of patients colonized or infected with carbapenem-resistant *Acinetobacter* [65,72]. Thoma et al. in their systematic review described waves of *Acinetobacter* spp. infections during the COVID-19 pandemic (between December 2019 and March 2021) [73]. This so-called “small pandemic” within the broader COVID-19 pandemic was considered a serious concern and potentially an even greater threat to COVID-19 ICU patients [66,70,71,74,75].

A major strength of this study is its large sample size, which enhances the statistical power and generalizability of the findings. Additionally, the inclusion of patients infected during the second, third, and fourth waves of the COVID-19 pandemic in Greece allowed for the analysis of disease progression across different SARS-CoV-2 variants. However, the results are subject to certain methodological limitations. Firstly, undiagnosed cases of diabetes mellitus (DM) may have been misclassified and included in the non-diabetic group, potentially leading to misclassification bias. Secondly, due to the overwhelming burden of admissions during the pandemic, it was not feasible to obtain detailed personal medical histories. As a result, important diabetes-related variables—such as disease duration, glycemic control status, HbA1c levels, and the presence of chronic diabetes-related complications—were not recorded. Finally, data on antimicrobial and antifungal treatments were not available in this study. Therefore, the potential impact of therapeutic interventions on patient outcomes, including mortality, could not be assessed. Another important factor that warrants consideration is the potential influence of tracheostomy on patient outcomes. Tracheostomy can significantly affect mortality rates, the duration of mechanical ventilation, the length of ICU stay, and particularly the incidence of ventilator-associated pneumonia (VAP), which is a major concern in critically ill COVID-19 patients. Recent studies have emphasized the importance of timing and practice of tracheostomy in ICU patients and its implications for clinical outcomes [76,77].

## 5. Conclusions

In this study of critically ill, intubated COVID-19 patients, DM was not found to be independently associated with ICU length of stay, 28-day ICU mortality, or the occurrence of MDR-GNB. These findings are consistent with the so-called “diabetes paradox”, which suggests that in the ICU setting, the presence of DM may not significantly influence outcomes, likely due to the overriding impact of acute illness severity. While DM remains a major risk factor for morbidity and mortality in the general population, its prognostic value in critically ill patients probably appears limited. This highlights the complexity of outcome determinants in the ICU and underscores the need for further research to better understand the interplay between chronic comorbidities and critical illness.

## Figures and Tables

**Table 1:** Demographic characteristics, clinical characteristics, comorbidities, prior medications, and laboratory markers of COVID-19 ICU patients with T2DM (LOS ≤ 28 days) by 28-day ICU mortality.

	Total (*n* = 86)	Deceased (*n* = 62)	Survived (*n* = 24)	*p*-Value
Age (years), Mean ± SD	69.1 (10.2)	71.6 (6.6)	62.8 (14.5)	0.002
Gender (male), *n*(%)	43 (50.0%)	34 (54.8%)	9 (37.5%)	0.149
Pandemic Wave, *n*(%)				
2nd	21 (24.4%)	14 (22.6%)	7 (29.2%)	0.487
3rd	39 (45.3%)	27 (43.5%)	12 (50.0%)	
4th	26 (30.2%)	21 (33.9%)	5 (20.8%)	
LOS (days), mean ± SD	13.3 (7.4)	12.8 (7.2)	14.5 (8.0)	0.396
ICU Readmission, *n*(%)	5 (5.8%)	3 (4.8%)	2 (8.3%)	0.534
DLP, *n*(%)	67 (77.9%)	48 (77.4%)	19 (79.2%)	0.861
HTN, *n*(%)	61 (70.9%)	46 (74.2%)	15 (62.5%)	0.284
CAD, *n*(%)	19 (22.1%)	16 (25.8%)	3 (12.5%)	0.182
Arrhythmia, *n*(%)	10 (11.6%)	6 (9.7%)	4 (16.7%)	0.364
COPD, *n*(%)	5 (5.8%)	4 (6.5%)	1 (4.2%)	0.685
Cancer, *n*(%)	2 (2.3%)	1 (1.6%)	1 (4.2%)	0.481
Hypothyroidism, *n*(%)	11 (12.8%)	7 (11.3%)	4 (16.7%)	0.503
Hyperuricemia, *n*(%)	11 (12.8%)	9 (14.5%)	2 (8.3%)	0.441
Anemia *n*(%)	10 (11.6%)	8 (12.9%)	2 (8.3%)	0.553
BPH, *n*(%)	7 (8.1%)	6 (9.7%)	1 (4.2%)	0.402
Psychiatric Disorder, *n*(%)	16 (18.6%)	13 (21.0%)	3 (12.5%)	0.365
Statin, *n*(%)	59 (68.6%)	43 (69.4%)	16 (66.7%)	0.810
ASA, *n*(%)	32 (37.2%)	22 (35.5%)	10 (41.7%)	0.595
Clopidogrel, *n*(%)	15 (17.4%)	13 (21.0%)	2 (8.3%)	0.166
NOACs, *n*(%)	9 (10.5%)	7 (11.3%)	2 (8.3%)	0.688
Acenocoumarol, *n*(%)	1 (1.2%)	0 (0.0%)	1 (4.2%)	0.106
ARBs, *n*(%)	44 (51.2%)	32 (51.6%)	12 (50.0%)	0.893
Diuretics, *n*(%)	43 (50.0%)	31 (50.0%)	12 (50.0%)	1.000
CCBs, *n*(%)	34 (39.5%)	26 (41.9%)	8 (33.3%)	0.464
Beta-blockers *n*(%)	31 (36.0%)	22 (35.5%)	9 (37.5%)	0.861
ACEi, *n*(%)	9 (10.5%)	8 (12.9%)	1 (4.2%)	0.235
Aldosterone Antagonists, *n*(%)	4 (4.7%)	4 (6.5%)	0 (0.0%)	0.203
Central α-agonists, *n*(%)	4 (4.7%)	3 (4.8%)	1 (4.2%)	0.894
No Antidiabetic Treatment, *n*(%)	7 (8.1%)	2 (3.2%)	5 (20.8%)	0.021
OAD Monotherapy, *n*(%)	54 (62.8%)	44 (71.0%)	10 (41.7%)	
Insulin Monotherapy, *n*(%)	14 (16.3%)	9 (14.5%)	5 (20.8%)	
Combination OADs/Insulin, *n*(%)	11 (12.8%)	7 (11.3%)	4 (16.7%)	
MET, *n*(%)	54 (62.8%)	40 (64.5%)	14 (58.3%)	0.595
DPP-4i, *n*(%)	31 (36.0%)	24 (38.7%)	7 (29.2%)	0.408
SGLT-2i, *n*(%)	20 (23.3%)	13 (21.0%)	7 (29.2%)	0.420
SU *n*(%)	16 (18.6%)	12 (19.4%)	4 (16.7%)	0.774
GLP-1 RA, *n*(%)	9 (10.5%)	7 (11.3%)	2 (8.3%)	0.688
PIOn(%)	2 (2.3%)	2 (3.2%)	0 (0.0%)	0.517
APACHE II on Admission, Median (IQR)	14 (6)	14.5 (7)	13 (4)	0.010
AKI on Admission, *n*(%)	25 (29.1%)	22 (35.5%)	3 (12.5%)	0.035
Admission Glucose Value (mg/dL) mean ± SD	219.1 (84.8)	211.0 (75.8)	240.1 (103.4)	0.277
Mean Fasting Glucose (mg/dL) Mean ± SD	200.2 (48.8)	199.7 (45.4)	201.2 (57.8)	0.725
WBC (K/μL), Mean ± SD	14.4 (6.4)	15.3 (6.5)	12.1 (5.3)	0.033
Hct (%), Mean ± SD	36.1 (5.5)	36.3 (5.6)	35.7 (5.3)	0.473
Cr serum (mg/dL), Mean ± SD	1.4 (1.3)	1.5 (1.4)	1.1 (0.6)	0.045
eGFR (mL/min), Mean ± SD	62.1 (28.5)	58.2 (28.0)	72.1 (27.7)	0.053
Troponin (pg/mL), Mean ± SD	59.2 (124.3)	70.7 (152.1)	36.3 (21.6)	0.371
CRP (mg/L), Mean ±SD	108.3 (79.3)	109.5 (82.4)	104.5 (70.7)	0.949
Ferritin (ng/mL), Mean ± SD	1203.5 (1848.1)	1280.4 (1684.2)	955.7 (2339.2)	0.005
PCT (ng/mL), Mean ± SD	1.5 (4.3)	1.8 (4.7)	0.2 (0.3)	0.030
D-dimer (μg/dL), Mean ± SD	6048.8 (8382.9)	6963.2 (9451.5)	3402.1 (2656.0)	0.634

COVID-19 = Coronavirus disease 2019; ICU = intensive care unit; T2DM = type 2 diabetes mellitus; SD = standard deviation; LOS = length of stay; DLP = dyslipidemia; HTN = hypertension; CAD = coronary artery disease; COPD = chronic obstructive pulmonary disease; HUA = hyperuricemia; BPH = benign prostatic hyperplasia; ASA = acetylsalicylic acid; NOACs = non–vitamin K oral anticoagulants; ARBs = angiotensin receptor blockers; CCBs = calcium channel blockers; ACEi; = angiotensin-converting enzyme inhibitors; central α-agonists = centrally acting antihypertensives; OADs = oral antidiabetic drugs; MET = metformin; DPP-4i = dipeptidyl peptidase-4 inhibitors; SGLT-2i = sodium-glucose co-transporter-2 inhibitors; SU = sulfonylureas; GLP-1 RA = glucagon-like peptide-1 receptor agonists; PIO = pioglitazone; AKI = acute kidney injury; WBCs = white blood cells; Hct = hematocrit; Cr = creatinine; e-GFR = estimated glomerular filtration rate; CRP = C-reactive protein; PCT = procalcitonin.

**Table 2:** Multivariate logistic regression analysis for 28-day ICU mortality in all COVID-19 patients and in those with diabetes (LOS ≤ 28 days).

	All Patients (LOS ≤ 28 Days) Multivariate OR (95% CI)	*p*-Value	DM Patients (LOS ≤ 28 Days) Multivariate OR (95% CI)	*p*-Value
Age (years)	1.01 (0.98–1.04)	0.418	1.10 (1.02–1.18)	0.011
DM	1.12 (0.52–2.41)	0.769	-	-
HTN	1.01 (0.55–1.85)	0.980	-	-
CAD	1.82 (0.75–4.43)	0.184	1.97 (0.69–3.51)	0.497
COPD	2.85 (1.06–7.68)	0.038	-	-
APACHE II	1.14 (1.02–1.26)	0.017	1.02 (0.88–1.20)	0.763
AKI	1.56 (0.69–3.51)	0.283	4.63 (1.02–20.94)	0.047
Admission glucose value (mg/dL)	1.03 (0.99–1.07)	0.213	1.01 (0.99–1.12)	0.904
WBC (K/μL)	1.05 (1.00–1.09)	0.035	1.09 (0.98–1.20)	0.084
Ferritin (ng/mL)	1.00 (1.00–1.00)	0.058	-	-

COVID-19 = Coronavirus disease 2019; ICU = intensive care unit; DM = diabetes mellitus; LOS = length of stay; HTN = hypertension; CAD = coronary artery disease; COPD = chronic obstructive pulmonary disease; AKI = acute kidney injury; WBCs = white blood cells.

**Table 3:** Association between DM and MDR-GNB isolated from cultures of COVID-19 ICU patients.

		DM vs. Non-DM	95% CI		
Culture Type	Pathogen	OR	Lower 95%	Upper 95%	*p*-Value
Bronchial Secretion	Acinetobacter baumannii	2.179	1.397	3.399	<0.001
	Klebsiella pneumoniae	0.968	0.544	1.72	0.911
	Pseudomonas aeruginosa	0.661	0.328	1.332	0.247
	Stenotrophomonas maltophilia	0.986	0.426	2.284	0.974
	Enterobacter cloacae	2.761	0.549	13.888	0.218
	Enterobacter aerogenes	0.904	0.093	8.781	0.931
	Providencia stuartii	0.904	0.093	8.781	0.931
Blood	Acinetobacter baumannii	1.226	0.737	2.037	0.432
	Klebsiella pneumoniae	0.989	0.579	1.689	0.967
	Pseudomonas aeruginosa	0.556	0.185	1.67	0.295
	Providencia stuartii	0.382	0.046	3.142	0.371
	Stenotrophomonas maltophilia	0.382	0.046	3.142	0.371
CVC Tip	Acinetobacter baumannii	0.718	0.332	1.552	0.399
	Klebsiella pneumoniae	0.558	0.225	1.386	0.209
	Pseudomonas aeruginosa	1.168	0.297	4.596	0.824
	Providencia stuartii	0.673	0.141	3.217	0.620
Urine	Klebsiella pneumoniae	0.956	0.367	2.488	0.926
	Acinetobacter baumannii	0.789	0.284	2.191	0.649
	Providencia stuartii	1.087	0.208	5.686	0.921

COVID-19 = Coronavirus disease 2019; ICU = intensive care unit; DM = diabetes mellitus; MDR-GNB = multidrug-resistant Gram-negative bacteria; CVC = central venous catheter.

**Table 4:** Multivariate logistic regression analysis for predictors for *Acinetobacter baumannii* (bronchial secretions).

	*Acinetobacter baumannii* (Bronchial Secretions) Multivariate OR	95% CI (Lower–Upper)	*p*-Value
DM	2.046	1.256–3.333	0.004
DLP	1.15	0.733–1.803	0.543

DM = diabetes mellitus; DLP = dyslipidemia.

## Data Availability

The data that support the findings of this study are available on request from the corresponding author.

## Supplementary Materials

The following supporting information can be downloaded at https://www.mdpi.com/article/10.3390/diagnostics15101190/s1; Table S1: Demographic, clinical characteristics, comorbidities, prior medications, and laboratory markers of COVID-19 ICU patients with and without T2DM; Table S2: Prevalence of MDR-GNB in cultures of intubated COVID-19 ICU patients with and without DM; Table S3: Prevalence of MDR-GNB in cultures of intubated COVID-19 ICU patients with and without DM; Table S4: Univariate logistic regression analysis for predictors for Acinetobacter baumannii (bronchial secretions); Table S5: Univariate logistic regression analysis for association of MDR-GNB with overall mortality in COVID-19 intubated patients with DM.
