# Supplementary material for: Diabetes Mellitus and Multidrug-Resistant Gram-Negative Bacterial Infections in Critically Ill COVID-19 Patients: A Retrospective Observational Study
Source: Diagnostics (Basel). 2025 May 8;15(10):1190. doi: 10.3390/diagnostics15101190 (PMC12110607; doi:10.3390/diagnostics15101190)
Supplement: Supplementary file 1 [file diagnostics-15-01190-s001.zip › diagnostics-3600808-supplementary.pdf]

SUPPLEMENTAL MATERIAL

Diabetes mellitus and MDR Gram-negative bacterial infections in critically ill COVID-19 patients: A retrospective observational study

Table S1. Demographic, clinical characteristics, comorbidities, prior medications, and laboratory markers of COVID-19 ICU patients with and without T2DM.

	**Total (N = 416)**	**T2DM (n = 112)**	**Non-T2DM (n = 304)**	**p-value**
Age (years), mean ± SD	63.4 ± 12.5	68.8 ± 9.6	61.4 ± 12.8	< 0.001
Gender (male), n (%)	261 (62.7%)	59 (52.7%)	202 (66.4%)	0.010
Pandemic wave, n (%)				
2nd	97 (23.3%)	24 (21.4%)	73 (24.0%)	
3rd	117 (28.1%)	45 (40.2%)	72 (23.7%)	0.003
4th	202 (48.6%)	43 (38.4%)	159 (52.3%)	
LOS (days), mean ± SD	18.6 ± 13.8	20.0 ± 16.0	18.1 ± 12.9	0.493
28-day ICU mortality, n (%)	216 (51.9%)	62 (55.4%)	154 (50.7%)	0.395
Readmission, n (%)	26 (6.3%)	6 (5.4%)	20 (6.6%)	0.648
DLP, n (%)	188 (45.2%)	88 (78.6%)	100 (32.9%)	< 0.001
HTN, n (%)	206 (49.5%)	80 (71.4%)	126 (41.4%)	< 0.001
CAD, n (%)	52 (12.5%)	25 (22.3%)	27 (8.9%)	< 0.001
Arrhythmia, n (%)	40 (9.6%)	11 (9.8%)	29 (9.5%)	0.931
COPD, n (%)	34 (8.2%)	5 (4.5%)	29 (9.5%)	0.094
Cancer, n (%)	12 (2.9%)	4 (3.6%)	8 (2.6%)	0.611
Hypothyroidism, n (%)	39 (9.4%)	14 (12.5%)	25 (8.2%)	0.184
HUA, n (%)	26 (6.3%)	14 (12.5%)	12 (3.9%)	0.001
Anemia, n (%)	29 (7%)	13 (11.6%)	16 (5.3%)	0.024
BPH, n (%)	43 (10.3%)	10 (8.9%)	33 (10.9%)	0.567
Psychiatric disease, n (%)	65 (15.6%)	19 (17.0%)	46 (15.1%)	0.648
Statin, n (%)	164 (39.4%)	79 (70.5%)	85 (28.0%)	< 0.001
ASA, n (%)	73 (17.5%)	40 (35.7%)	33 (10.9%)	< 0.001
Clopidogrel, n (%)	41 (9.9%)	20 (17.9%)	21 (6.9%)	< 0.001
NOAC, n (%)	20 (4.8%)	10 (8.9%)	10 (3.3%)	0.017
Acenocoumarol, n (%)	6 (1.4%)	1 (0.9%)	5 (1.6%)	0.568
ARBs, n (%)	124 (29.8%)	55 (49.1%)	69 (22.7%)	< 0.001
Diuretics, n (%)	107 (25.7%)	51 (45.5%)	56 (18.4%)	< 0.001
Beta-blockers, n (%)	120 (28.8%)	43 (38.4%)	77 (25.3%)	0.009
CCBs, n (%)	104 (25.0%)	42 (37.5%)	62 (20.4%)	< 0.001
ACEi, n (%)	38 (9.1%)	13 (11.6%)	25 (8.2%)	0.288
Aldosterone antagonists, n (%)	7 (1.7%)	5 (4.5%)	2 (0.7%)	0.007
Central α-agonists, n (%)	11 (2.6%)	5 (4.5%)	6 (2.0%)	0.160
APACHE II on admission, median (IQR)	13 (4)	14 (4)	12 (3)	< 0.001
AKI on admission, n (%)	74 (17.9%)	29 (25.9%)	45 (14.9%)	0.010
Admission glucose value (mg/dL), mean ± SD	176.8 ± 66.6	213.5 ± 80.5	163.2 ± 54.7	< 0.001

Mean fasting glucose (mg/dL), mean ± SD	158.1 ± 47.1	197.3 ± 46.1	143.5 ± 38.3	< 0.001
WBC (K/μL), mean ± SD	14.4 ± 7.8	13.6 ± 6.3	14.7 ± 8.3	0.408
Hct (%), mean ± SD	36.4 ± 5.7	35.8 ± 5.4	36.5 ± 5.8	0.181
Cr serum (mg/dL), mean ± SD	1.1 ± 0.8	1.3 ± 1.1	1.0 ± 0.7	0.003
eGFR (mL/min), mean ± SD	77.5 ± 29.3	66.3 ± 28.8	81.6 ± 28.5	< 0.001
Troponin (pg/mL), mean ± SD	458.7 ± 2159.7	99.2 ± 226.9	583.7 ± 2496.3	0.993
CRP (mg/L), mean ± SD	127.1 ± 96.2	107.3 ± 76.8	134.2 ± 101.4	0.067
Ferritin (ng/mL), mean ± SD	1184.9 ± 1856.5	1166.5 ± 1733.6	1191.7 ± 1902.4	0.211
PCT (ng/mL), mean ± SD	1.6 ± 5.8	1.1 ± 3.6	1.7 ± 6.4	0.137
D-dimer (μg/dL), mean ± SD	6005.7 ± 8599.6	5710.4 ± 8167.2	6106.9 ± 8754.2	0.683

*COVID-19=Coronavirus disease 2019, ICU=Intensive Care Unit, T2DM=type 2 Diabetes Mellitus, SD=standard Deviation LOS=Length of Stay, DLP=Dyslipidemia, HTN=Hypertension, CAD=Coronary Artery Disease, COPD=Chronic Obstructive Pulmonary Disease, HUA=Hyperuricemia, BPH=Benign Prostatic Hyperplasia, ASA=Acetylsalicylic acid, NOAC=Non–Vitamin K Oral Anticoagulants, ARBs=Angiotensin Receptor Blockers, CCBs=Calcium Channel Blockers, ACEi,=Angiotensin-Converting Enzyme Inhibitors, Central α-agonists=Centrally Acting Antihypertensives, AKI=Acute Kidney Injury, WBC=White Blood Cells, Hct=Hematocrit, Cr=Creatinine, eGFR=Estimated Glomerular Filtration Rate, CRP=C-Reactive Protein, PCT=Procalcitonin

Table S2. Prevalence of MDR-GNB in cultures of intubated COVID-19 ICU patients with and without DM

Culture Type	Pathogen	Total n (%)	DM n (%)	Non-DM n (%)	p-value
Bronchial Secretion	Acinetobacter baumannii	145 (34.9%)	54 (48.2%)	91 (29.9%)	<0.001
	Klebsiella pneumoniae	72 (17.3%)	19 (17.0%)	53 (17.4%)	0.911
	Pseudomonas aeruginosa	54 (13.0%)	11 (9.8%)	43 (14.1%)	0.245
	Stenotrophomonas maltophilia	30 (7.2%)	8 (7.1%)	22 (7.2%)	0.974
	Enterobacter cloacae	6 (1.4%)	3 (2.7%)	3 (1.0%)	0.199
	Enterobacter aerogenes	4 (1.0%)	1 (0.9%)	3 (1.0%)	0.931
	Providencia stuartii	4 (1.0%)	1 (0.9%)	3 (1.0%)	0.931
	Proteus mirabilis	1 (0.2%)	0 (0%)	1 (0.3%)	0.543
	Klebsiella oxytoca	1 (0.2%)	0 (0%)	1 (0.3%)	0.543
Blood	Acinetobacter baumannii	93 (22.4%)	28 (25.0%)	65 (21.4%)	0.432
	Klebsiella pneumoniae	86 (20.7%)	23 (20.5%)	63 (20.7%)	0.967
	Pseudomonas aeruginosa	23 (5.5%)	4 (3.6%)	19 (6.3%)	0.289
	Providencia stuartii	8 (1.9%)	1 (0.9%)	7 (2.3%)	0.353
	Stenotrophomonas maltophilia	8 (1.9%)	1 (0.9%)	7 (2.3%)	0.353
	Enterobacter cloacae	2 (0.5%)	0 (0%)	2 (0.7%)	0.390
	Enterobacter aerogenes	1 (0.2%)	0 (0%)	1 (0.3%)	0.543
	Achromobacter	1 (0.2%)	0 (0%)	1 (0.3%)	0.543
	Klebsiella oxytoca	1 (0.2%)	0 (0%)	1 (0.3%)	0.543
CVC Tip	Acinetobacter baumannii	42 (10.1%)	9 (8.0%)	33 (10.9%)	0.397
	Klebsiella pneumoniae	34 (8.2%)	6 (5.4%)	28 (9.2%)	0.203
	Pseudomonas aeruginosa	10 (2.4%)	3 (2.7%)	7 (2.3%)	0.824
	Providencia stuartii	10 (2.4%)	2 (1.8%)	8 (2.6%)	0.617
	Enterobacter cloacae	1 (0.2%)	0 (0%)	1 (0.3%)	0.543
	Stenotrophomonas maltophilia	2 (0.5%)	0 (0%)	2 (0.7%)	0.390
Urine	Klebsiella pneumoniae	23 (5.5%)	6 (5.4%)	17 (5.6%)	0.926
	Acinetobacter baumannii	22 (5.3%)	5 (4.5%)	17 (5.6%)	0.648
	Providencia stuartii	7 (1.7%)	2 (1.8%)	5 (1.6%)	0.921
	Pseudomonas aeruginosa	4 (1.0%)	0 (0%)	4 (1.3%)	0.223
	Escherichia coli	1 (0.2%)	1 (0.9%)	0 (0%)	0.099

*COVID-19=Coronavirus disease 2019, ICU=Intensive Care Unit, MDR-GNB=Multidrug-resistant Gram-negative Bacteria, DM=Diabetes Mellitus, CVC=Central Venous Catheter

Table S3. Univariate logistic regression analysis for 28-day ICU mortality among all COVID-19 patients and patients with DM (LOS≤28 Days)

	**All Patients (LOS≤28 Days) Univariate OR (95% CI)**	**p-value**	**DM Patients (LOS≤28 Days) Univariate OR (95% CI)**	**p-value**
Age (years)	1.06 (1.04–1.08)	<0.001	1.10 (1.04–1.16)	0.002
Gender (male)	1.45 (0.92–2.29)	0.111	2.02 (0.77–5.32)	0.153
DM	1.59 (0.93–2.72)	0.088	-	-
DLP	1.35 (0.86–2.12)	0.196	0.90 (0.29–2.85)	0.861
HTN	1.88 (1.19–2.96)	0.007	1.73 (0.63–4.70)	0.287
CAD	2.36 (1.09–5.10)	0.029	2.44 (0.64–9.27)	0.192
Arrhythmia	0.67 (0.33–1.37)	0.272	0.54 (0.14–2.10)	0.370
COPD	2.85 (1.06–7.68)	0.038	1.59 (0.17–14.96)	0.687
Cancer	1.96 (0.40–9.59)	0.406	0.38 (0.02–6.28)	0.497
Hypothyroidism	1.30 (0.60–2.83)	0.510	0.64 (0.17–2.41)	0.506
HUA	1.70 (0.60–4.80)	0.316	1.87 (0.37–9.35)	0.447
Anemia	1.95 (0.70–5.42)	0.202	1.63 (0.32–8.29)	0.556
BPH	1.34 (0.64–2.83)	0.436	2.46 (0.28–21.63)	0.416
Psychiatric Disorder	1.09 (0.59–1.99)	0.785	1.86 (0.48–7.20)	0.371
APACHE II Score	1.24 (1.15–1.35)	<0.001	1.21 (1.04–1.41)	0.013
AKI	2.92 (1.49–5.72)	0.002	3.85 (1.03–14.37)	0.045
Admission glucose value (mg/dL)	1.00 (0.99–1.00)	0.715	0.99 (0.99–1.00)	0.155
Mean fast glucose (mg/dL)	1.01 (1.00–1.01)	0.007	0.99 (0.99–1.01)	0.904
WBC (K/μL)	1.07 (1.03–1.12)	<0.001	1.10 (1.00–1.19)	0.041
Hct (%)	1.03 (0.99–1.07)	0.213	1.02 (0.93–1.12)	0.659
Cr serum (mg/dL)	1.76 (1.14–2.72)	0.010	1.88 (0.76–4.65)	0.171
eGFR (mL/min)	0.98 (0.98–0.99)	<0.001	0.98 (0.97–1.00)	0.047
Troponin (pg/mL)	1.00 (0.99–1.01)	0.139	1.00 (0.99–1.02)	0.589
CRP (mg/L)	1.00 (1.00–1.00)	0.110	1.00 (0.99–1.01)	0.811
Ferritin (ng/mL)	1.00 (1.00–1.00)	0.004	1.00 (1.00–1.00)	0.520
PCT (ng/mL)	1.03 (0.97–1.10)	0.302	6.43 (0.18–236.1)	0.312
D-dimer (μg/dL)	1.00 (1.00–1.00)	0.191	1.00 (1.00–1.00)	0.145

*COVID-19=Coronavirus disease 2019, ICU=Intensive Care Unit, T2DM=type 2 Diabetes Mellitus, LOS=Length of Stay, DLP=Dyslipidemia, HTN=Hypertension, CAD=Coronary Artery Disease, COPD=Chronic Obstructive Pulmonary Disease, HUA=Hyperuricemia, BPH=Benign Prostatic Hyperplasia, AKI=Acute Kidney Injury, WBC=White Blood Cells, Hct=Hematocrit, Cr=Creatinine, eGFR=Estimated Glomerular Filtration Rate, CRP=C-Reactive Protein, PCT=Procalcitonin

Table S4. Univariate logistic regression analysis for predictors for *Acinetobacter baumannii* (bronchial secretions)

	***Acinetobacter baumannii* (bronchial secretions) Univariate OR**	**95% CI (Lower–Upper)**	**p-value**
Age (years)	1.006	0.990–1.023	0.446
Gender (Male)	1.048	0.690–1.591	0.827
DM	2.179	1.397–3.399	<0.001
DLP	1.498	0.999–2.247	0.051
HTN	1.098	0.733–1.643	0.651
CAD	1.195	0.656–2.175	0.560
Arrhythmia	0.783	0.386–1.590	0.499
COPD	1.021	0.490–2.128	0.955
Cancer	0.365	0.079–1.689	0.197
Hypothyroidism	1.052	0.529–2.092	0.886
HUA	0.989	0.429–2.277	0.979
Anemia	1.570	0.733–3.361	0.246
BPH	0.892	0.455–1.747	0.739
Psychiatric Disorder	1.028	0.591–1.788	0.922
APACHE II	0.993	0.944–1.045	0.794
AKI	1.160	0.689–1.952	0.576
Admission glucose value (mg/dL)	1.002	0.999–1.005	0.259
Mean fasting glucose (mg/dL)	1.002	0.997–1.006	0.425
WBC (K/μL)	0.975	0.948–1.003	0.076
Hct (%)	1.016	0.978–1.055	0.417
Cr serum (mg/dL)	0.959	0.744–1.238	0.750
eGFR (mL/min)	0.997	0.990–1.004	0.402
Troponin (pg/mL)	1.000	1.000–1.000	0.233
CRP (mg/L)	1.001	0.998–1.003	0.584
Ferritin (ng/mL)	1.000	1.000–1.000	0.923
PCT (ng/mL)	1.018	0.977–1.061	0.396
D-dimer (μg/dL)	1.000	1.000–1.000	0.123

*DM=Diabetes Mellitus, DLP=Dyslipidemia, HTN=Hypertension, CAD=Coronary Artery Disease, COPD=Chronic Obstructive Pulmonary Disease, HUA=Hyperuricemia, BPH=Benign Prostatic Hyperplasia, AKI=Acute Kidney Injury, WBC=White Blood Cells, Hct=Hematocrit, Cr=Creatinine, eGFR=Estimated Glomerular Filtration Rate, CRP=C-Reactive Protein, PCT=Procalcitonin

Table S5. Univariate logistic regression analysis for association of MDR-GNB with overall mortality in COVID-19 intubated patients with DM

Pathogen	Overall mortality DM Univariate OR	95% CI (Lower–Upper)	p-value
Acinetobacter baumannii (Bronchial Secretions)	0.985	0.437–2.220	0.970
Klebsiella pneumoniae (Bronchial Secretions)	0.665	0.236–1.875	0.441
Pseudomonas aeruginosa (Bronchial Secretions)	1.993	0.407–9.767	0.395
Stenotrophomonas maltophilia (Bronchial Secretions)	3.111	0.367–26.344	0.298
Enterobacter cloacae (Bronchial Secretions)	0.831	0.073–9.494	0.882
Acinetobacter baumannii (Blood)	0.679	0.273–1.686	0.404
Klebsiella pneumoniae (Blood)	3.390	0.933–12.322	0.064
Pseudomonas aeruginosa (Blood)	0.403	0.054–2.986	0.374
Acinetobacter baumannii (CVC Tip)	0.490	0.123–1.953	0.312
Klebsiella pneumoniae (CVC Tip)	2.162	0.243–19.256	0.489
Providencia stuartii (CVC Tip)	0.410	0.025–6.761	0.533
Klebsiella pneumoniae (Urine)	0.395	0.075–2.066	0.271
Acinetobacter baumannii (Urine)	0.612	0.097–3.842	0.271
Providencia stuartii (Urine)	0.410	0.025–6.761	0.533

*COVID-19=Coronavirus disease 2019, MDR-GNB=Multidrug-resistant Gram-negative Bacteria, DM=Diabetes Mellitus, CVC=Central Venous Catheter
